# In Vitro Assessment of Osteogenic Modulation and Molecular Responses Induced by Contemporary Endodontic Sealers in MC3T3-E1 Pre-Osteoblasts

**DOI:** 10.3390/dj14030160

**Published:** 2026-03-11

**Authors:** Yuka Miyamoto, Yuka Kato, Ryan Needle, Julie Yongsook Kim, Jin Koo Kim, Paul H. Krebsbach, Insoon Chang

**Affiliations:** 1 Section of Endodontics, UCLA School of Dentistry, Los Angeles, CA 90095, USA; miyamoto@dent.asahi-u.ac.jp (Y.M.);; 2 Department of Oral Pathology, School of Dentistry, Asahi University, Gifu 501-0296, Japan; 3 Department of Restorative and Biomaterials Sciences, School of Dentistry, Meikai University, Sakado 350-0248, Japan; 4 Section of Restorative Dentistry, UCLA School of Dentistry, Los Angeles, CA 90095, USA; 5 Division of Oral and Systemic Health Sciences, UCLA School of Dentistry, Los Angeles, CA 90095, USA; kjkkribb@ucla.edu (J.K.K.); pkrebsbach@dentistry.ucla.edu (P.H.K.)

**Keywords:** endodontic sealers, MAPK pathway, periapical tissue response, cell–material interactions, osteogenic-associated responses, bioceramic sealer

## Abstract

**Background/Objectives**: Endodontic sealers can interact with periapical tissues through extrusion, yet the molecular mechanisms underlying their biological effects remain poorly defined. This study investigated how commonly used sealers influence mitogen-activated protein kinase (MAPK) signaling, cell viability, and osteogenic-associated responses in MC3T3-E1 pre-osteoblasts. **Methods**: Four commercial sealers, Calcium-silicate-based Bioceramic Sealer (EndoSequence^®^ BC Sealer, BC), Zinc oxide eugenol sealer (Kerr Pulp Canal Sealer, ZOE), Sealapex™, and AH26^®^, were applied as standardized pellets, allowed to set, and cultured with MC3T3-E1 cells. Calcium deposition was assessed by Alizarin Red S (ARS) staining, and MAPK activation was evaluated by Western blotting. Due to excessive solubility (Sealapex™) or poor cell survival (AH26^®^), mechanistic analyses were performed only for BC and ZOE. Osteogenic-associated gene expression was measured by qRT-PCR, and the functional role of MAPK signaling was assessed using ERK, JNK, and p38 inhibitors. **Results**: BC and Sealapex™ produced robust ARS staining, while ZOE and AH26^®^ produced minimal mineral-associated staining. Both BC and ZOE activated ERK, JNK, and p38, with ZOE inducing higher phosphorylation. However, BC maintained greater cell viability and increased *Runx2* and *Osx* expression, whereas ZOE impaired early cell attachment and viability. MAPK inhibition in BC-treated cultures reduced osteogenic-associated gene expression and ARS staining, indicating MAPK involvement in BC-mediated responses. **Conclusions**: BC and ZOE elicit distinct MAPK activation patterns and cellular responses. Under the conditions tested, BC promoted a more favorable osteogenic-associated response, whereas ZOE compromised early cell viability. These mechanistic insights may help explain clinical differences in periapical tissue responses to sealer extrusion.

## 1. Introduction

Obturation is the final step of endodontic therapy and is a critical determinant of long-term clinical success [1,2]. Over the past several decades, diverse sealer formulations have been developed to optimize properties such as sealing ability, biocompatibility, and clinical handling [3,4]. Endodontic sealer extrusion is frequently observed during root canal obturation, particularly with warm vertical or carrier-based techniques, with reported rates approaching 60% of treated canals [5,6]. More recently, a systematic review and meta-analysis reported that sealer extrusion was associated with a 32% higher risk of non-healing events compared with cases without extrusion [7]. However, even in the absence of radiographic evidence of extrusion beyond the apex, sealers can still interact with periapical tissues, as materials set at or near the apical foramen may release soluble components or toxic by-products that diffuse through dentinal tubules, lateral canals, or apical microleakage, enabling direct biologic contact with surrounding tissues [6,8].

These direct sealer material–tissue interactions can significantly influence periapical healing by modulating cellular viability, inflammatory responses, and osteogenic or periodontal ligament cell differentiation, with biological outcomes largely dependent on the physicochemical properties of each sealer [4,9]. Calcium silicate-based bioceramic (BC) sealers, for example, generate an alkaline environment and release calcium ions that participate in hydroxyapatite formation and have been associated with favorable tissue responses [10,11]. In contrast, zinc oxide eugenol (ZOE)-based sealers have shown limited mineralization potential and variable cytocompatibility in vitro [4,9]. Despite the extensive clinical use of these materials, the biological behavior at the sealer–tissue interface is not well understood, particularly the mechanisms governing stress responses and osteogenic-associated phenotype. Although overt adverse reactions to sealer extrusion are relatively uncommon [4,9], clinical observations alone cannot elucidate the cellular or molecular events occurring at the tissue–material interface. A mechanistic understanding of how different sealer chemistries influence cell viability, stress-related signaling, and osteogenic-associated pathways is, therefore, essential, as it provides insight into biological effects that may not be clinically apparent and supports more evidence-based evaluation of materials.

The mitogen-activated protein kinase (MAPK) pathway is a key regulator of both cellular stress responses and osteogenic differentiation, integrating extracellular stimuli by activating ERK, JNK, and p38 to control processes such as cell survival, proliferation, and the expression of osteogenic transcription factors, including *Runx2* and *Osx* [12,13,14,15,16]. In this study, we examined the effects of commonly used endodontic sealers on MAPK activation, cell viability, and osteogenic-associated responses in the murine MC3T3-E1 pre-osteoblast cell line. Our goal was to elucidate the mechanisms that may contribute to the distinct biological behaviors reported for these materials. Understanding these mechanisms is essential for predicting tissue responses and informing safer, more biologically compatible sealer selection.

## 2. Materials and Methods

### 2.1. Sealers

The investigators independently purchased all sealers, and materials were selected to represent four major commercial sealer types commonly used in clinical practice [17]: AH26^®^ (epoxy resin-based; Dentsply Sirona, York, PA, USA), Pulp Canal Sealer™ (ZOE-based; Kerr Corporation, Orange, CA, USA), EndoSequence^®^ BC Sealer (calcium silicate-based; Brasseler USA, Savannah, GA, USA), and Sealapex™ (calcium hydroxide-based; Kerr). Experimental procedures were conducted in accordance with established protocols and guided by the general principles outlined in ISO 10993-12 [18] to ensure methodological consistency, reproducibility, and comparability among groups.

For pellet preparation, sterilized O-rings (3 mm inner diameter) were placed directly onto the surface of sterile 6-well plates (Corning Inc., Corning, NY, USA), and 10 μL of each freshly mixed sealer, prepared according to the manufacturer’s instructions, was dispensed into the O-ring to achieve standardized geometry and volume. The materials were allowed to fully set for 24 h at 37 °C under 5% CO_2_, as recommended by the manufacturers and in accordance with ISO 6876 [19], to minimize the influence of incomplete setting. After setting, the O-rings were carefully removed, and the sealer pellets were sterilized by UV irradiation for 30 min to reduce microbial contamination without thermal alteration. The plates were kept in the biosafety cabinet to maintain a sterile environment until cell seeding. This standardized direct-contact model provided uniform pellet size and surface area for consistent material–cell interaction and was selected to simulate potential exposure of periapical cells to extruded sealers under in vitro conditions, as described in previous biocompatibility studies of endodontic materials.

### 2.2. Cell Culture and Viability Assay

Murine MC3T3-E1 Subclone 4 pre-osteoblasts (ATCC CRL-2593™, ATCC, Manassas, VA, USA) were maintained in α-MEM supplemented with 10% fetal bovine serum and 1% penicillin/streptomycin at 37 °C in a humidified atmosphere containing 5% CO_2_. In vitro cytotoxicity testing was performed in accordance with the general principles of ISO 10993-5 [20]. For direct-contact cytotoxicity assays, 5 × 10^4^ cells were seeded per well in 6-well plates containing three pre-set, UV-sterilized sealer pellets (Section 2.1) in 3 mL of culture medium. Cell morphology was monitored daily by phase-contrast microscopy, and representative images were captured on days 4 and 5. On day 5, cells were harvested, and viability was assessed using trypan blue exclusion (1:1 dilution with 0.4% trypan blue), with viable and non-viable cells quantified using a hemocytometer.

### 2.3. Alizarin Red S (ARS) Staining

For ARS staining assays, 3 × 10^5^ cells were seeded per well in 6-well plates containing one or three pre-set, UV-sterilized sealer pellets in 3 mL of culture medium. At ~80% confluence, culture medium was switched to osteogenic induction medium (10 mM β-glycerophosphate, 50 µg/mL ascorbic acid, 100 nM dexamethasone). For ARS staining, cells were maintained in induction medium for 2 weeks, washed, fixed in 70% ethanol for 1 h, and stained with 1% ARS (Solarbio Life Sciences, Beijing, China). ARS quantification in ImageJ software (version 1.54d, National Institutes of health, Bethesda, MD, USA) involved converting images to 8-bit grayscale, measuring mean gray values of the stained regions, and inverting the values so that higher values reflected greater mineral deposition.

### 2.4. Quantitative Reverse Transcription PCR (qRT-PCR) and Western Blot

Total RNA was extracted using TRIzol (Invitrogen, Waltham, MA, USA) and reverse-transcribed with the SuperScript First-Strand Synthesis System. qRT-PCR was performed using SYBR Green supermix (Bio-Rad Laboratories, Hercules, CA, USA) on a CX96 system with a 45-cycle program. *Gapdh* served as internal control. Primer sequences were: *Runx2* (F: 5′-GACTGTGGTTACCGTCATGGC-3′, R: 5′-ACTTGGTTTTTCATAACAGCGGA-3′), *Sp7*/*Osx* (F: 5′-CCTCCTCAGCTCACCTTCTC-3′, R: 5′-GGGTAGTCATTTGCCTCCTC-3′), and *Gapdh* (F: 5′-ACAACTTTGGCATTGTGGAA-3′, R: 5′-GATGCAGGGATGATGTTCTG-3′). For Western blotting, whole-cell lysates were prepared using Protein Lysate (Sigma-Aldrich, St. Louis, MO, USA), separated on a 4–20% gradient tris-glycine gel, transferred to a PVDF membrane, blocked in 5% milk, and incubated with primary antibodies overnight at 4 °C. Primary antibodies purchased from Cell Signaling Technology were phospho (p)-Erk1/2 (1:2000), Erk1/2 (1:2000), p-p38 (1:2000), p38 (1:2000), p-c-Jun/Ser73 (1:2000), c-Jun (1:1000), Runx2 (1:2000), Gapdh (1:5000), and from Santa-Cruz Biotechnology was Osx (1:1000). Blots were incubated with HRP-conjugated secondary antibodies (Promega Corporation, Madison, WI, USA) for 1 h, and signals were detected using SuperSignal West Pico Chemiluminescence (Thermo Fisher Scientific, Waltham, MA, USA).

### 2.5. MAPK Inhibition

To assess MAPK pathway involvement, MC3T3-E1 cells were cultured with BC in the presence of MAPK inhibitors: SB203580 (p38 inhibitor, Sigma-Aldrich), SP600125 (Jnk inhibitor, Sigma-Aldrich), and U0126 (Erk inhibitor, Cell Signaling Technology, Danvers, MA, USA).

### 2.6. Calcium and pH Assay

Three pre-set and UV-sterilized sealer pellets were incubated in 6-well plates containing 3 mL of UltraPure^TM^ distilled water (Invitrogen) per well at 37 °C under 5% CO_2_. Calcium ion release into the water was measured from day 1 to day 4 using a Calcium Assay Kit (Sigma-Aldrich) and a BioTek microplate reader with Gen5 software (version 3.17.17, BioTak Instruments, Winooski, VT, USA) (absorbance at 612 nm) according to the manufacturer’s instructions. Sealer-induced pH changes were measured from day 1 to day 4 using MQuant Universal pH-indicator strips (pH 0–14, Sigma-Aldrich) and analyzed with the MQuant StripScan app (Sigma-Aldrich) according to the manufacturer’s instructions.

### 2.7. Statistical Analysis

All data are presented as mean ± standard deviation (SD). Statistical significance between two groups was assessed using an unpaired Student’s *t*-test, and comparisons among three or more groups were evaluated by one-way ANOVA with Tukey’s post hoc test. A *p*-value < 0.05 was considered statistically significant. Analyses were performed using GraphPad Prism 9.

## 3. Results

### 3.1. ARS Staining Revealed Distinct Sealer-Dependent Calcium-Associated Responses

**Figure 1:**
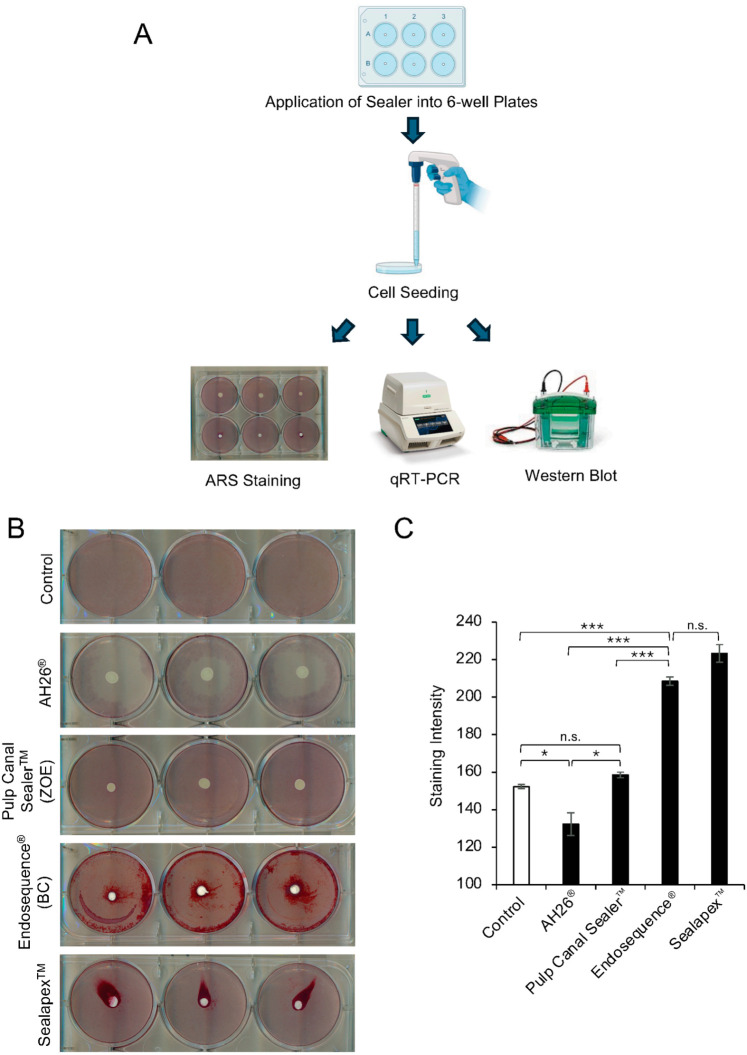
ARS staining and analysis of the effect of various endodontic sealers in MC3T3-E1 cells. (**A**) Schematic diagram of the experimental workflow. Sealers (10 μL spots, 3 mm in diameter) were placed at the center of 6-well plates, allowed to set for 24 h in a laminar flow hood, and sterilized by UV irradiation for 30 min before cell seeding. MC3T3-E1 pre-osteoblasts were then cultured and analyzed by ARS staining, qRT-PCR, and Western blotting. (**B**) Representative images of ARS staining of MC3T3-E1 cells cultured with various endodontic sealers: AH26^®^, ZOE, BC, and Sealapex™. (**C**) Quantification of ARS staining intensity using ImageJ (NIH). Data are presented as mean ± SD from triplicate assays, and the experiments were repeated three times. Statistical significance was assessed by one-way ANOVA with Tukey’s post hoc test (* *p* < 0.01, *** *p* < 0.0001, n.s., not significant).

Four sealers were evaluated for calcium-associated ARS staining and mRNA, protein, and phosphorylation levels of MAPK-related pathways (Figure 1A). BC and Sealapex™ produced strong ARS staining, whereas ZOE and AH26^®^ demonstrated minimal or no staining (Figure 1B). Quantitative analysis confirmed significantly higher ARS values for BC and Sealapex™ compared with the other groups, with no difference between them (Figure 1C). ZOE exhibited staining comparable to the control (no sealer), while AH26^®^ showed the lowest signal.

Time-dependent cytotoxicity analysis revealed a progressive increase in cell death for both AH26^®^ and Sealapex™. In contrast, BC and ZOE showed relatively low overall cell death; however, ZOE induced a transient increase in cell death at early time points, which decreased by day 4 (Supplementary Figure S1A). Spatial analysis demonstrated a markedly larger cell-free zone surrounding AH26^®^ pellets (approximately 9 mm in diameter) compared with the other groups (Figure 1B and Supplementary Figure S1B). Measurement of calcium ion release over 1–4 days showed significantly higher calcium concentrations in BC and Sealapex™ than in ZOE and AH26^®^ (Supplementary Figure S2A). These findings are consistent with prior reports of sustained calcium release and alkalinization by BC compared with ZOE-based sealers [21]. Additionally, increased pH levels were observed in cultures exposed to BC, ZOE, and Sealapex™ relative to the control (Supplementary Figure S2B).

For further data collection and analysis, although Sealapex™ showed marked calcium deposition in surrounding MC3T3-E1 cells, it was not included in subsequent mechanistic analyses due to progressive cell death and insufficient viable cells, as well as its high solubility, which resulted in loss of pellet integrity and compromised reliable cell isolation, with dissolved material co-localizing with harvested cells. AH26^®^ was also excluded because insufficient viable cells were available for downstream molecular analyses. Accordingly, BC and ZOE were selected for subsequent mechanistic investigation.

### 3.2. BC Enhanced ARS Staining and Osteogenic Marker Expression Compared with ZOE

**Figure 2:**
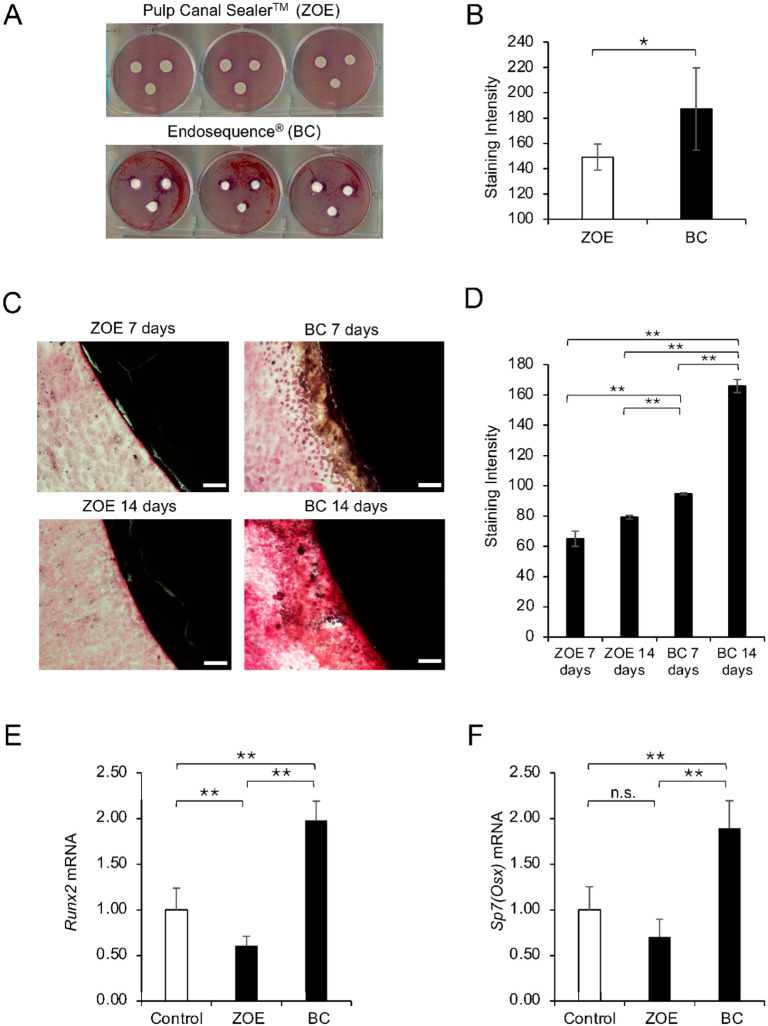
Osteogenic potential of ZOE and BC sealers in MC3T3-E1 cells. (**A**) Representative images of ARS staining of MC3T3-E1 cells cultured with ZOE or BC sealers. (**B**) Quantitative analysis of ARS staining intensity (ImageJ). Data are presented as mean ± SD from triplicate assays, and the experiments were repeated three times. Statistical significance was assessed by Student’s *t*-test (* *p* < 0.01). (**C**) Representative images of ARS staining of cells cultured with ZOE and BC for 7 and 14 days after osteogenic induction. Scale bars = 100 μm. (**D**) Quantitative analysis of ARS staining intensity at 7 and 14 days after switching to OI medium. Data are presented as mean ± SD from triplicate assays, and the experiments were repeated three times. Statistical significance was assessed by one-way ANOVA with Tukey’s post hoc test (** *p* < 0.001). (**E**) *Runx2* mRNA expression in cells cultured under control (no sealer), ZOE, and BC conditions for 1 week after switching to OI medium (qRT-PCR). Data are presented as mean ± SD from triplicate assays, and the experiments were repeated three times. Statistical significance was assessed by one-way ANOVA with Tukey’s post hoc test (** *p* < 0.001). (**F**) *Sp7 (Osx)* mRNA expression in cells cultured under control (no sealer), ZOE, and BC conditions for 1 week after switching to OI medium (qRT-PCR). Data are presented as mean ± SD from triplicate assays, and the experiments were repeated three times. Statistical significance was assessed by one-way ANOVA with Tukey’s post hoc test (** *p* < 0.001, n.s., not significant).

Stronger ARS staining was consistently observed in the BC group compared with the ZOE group (Figure 2A,B). In the time-course analysis, BC showed a significant increase in staining intensity from day 7 to day 14, whereas ZOE exhibited weak staining at both time points. BC at each time point also showed significantly higher staining intensity than ZOE (Figure 2C,D). qRT-PCR analysis demonstrated increased *Runx2* and *Sp7* (*Osx*) expression in the BC group (Figure 2E,F), while ZOE showed downregulation of *Runx2* and no significant change in *Sp7* compared with the control. Overall, BC-treated cells showed higher ARS staining and greater expression of early osteogenic genes than ZOE-treated cells in this study.

### 3.3. Differential MAPK Signaling and Cellular Response to BC and ZOE

**Figure 3:**
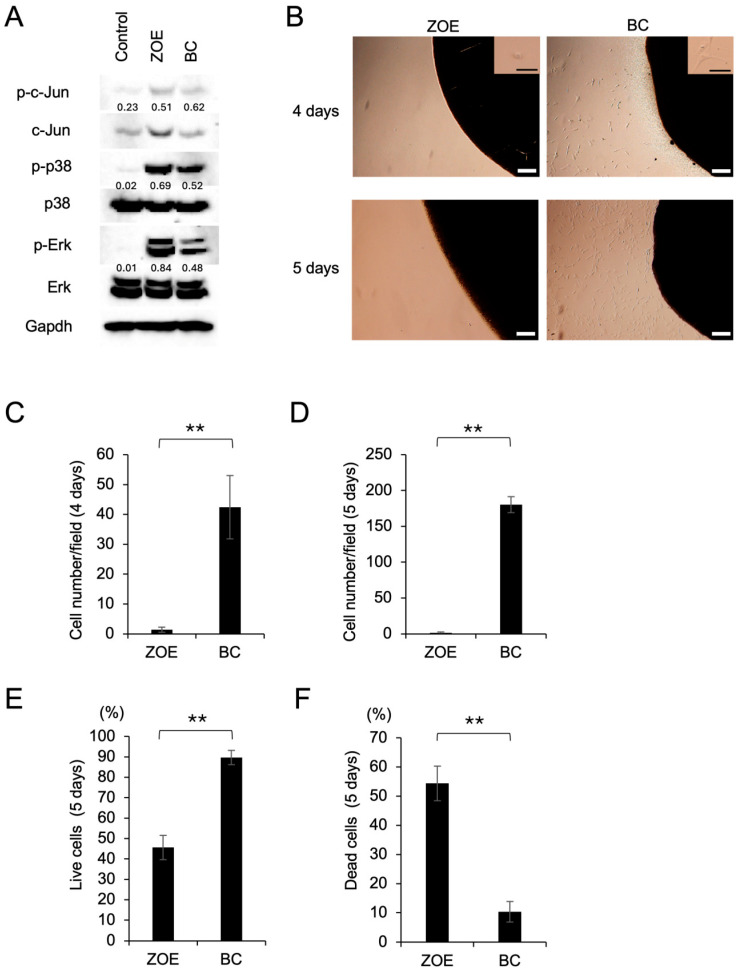
Effects of ZOE and BC sealers on MC3T3-E1 cell viability and MAPK signaling. (**A**) Western blot analysis of phosphorylated and total c-Jun, p38, and Erk in cells cultured with ZOE or BC for 1 week. Gapdh (loading control). Phospho-c-Jun, p38, and Erk band intensities were normalized to their respective total protein levels. (**B**) Representative images of MC3T3-E1 cells cultured in the presence of ZOE or BC for 4 and 5 days. Scale bars: 100 μm (white), 50 μm (black). (**C**,**D**) Quantification of cell numbers around sealer spots at day 4 (**C**) and day 5 (**D**). The number of cells in five random fields was counted per experimental condition under light microscopy. Data are presented as mean ± SD, and the experiments were repeated three times. Statistical significance was assessed by Student’s *t*-test (** *p* < 0.001). (**E**,**F**) Percentages of viable (**E**) and non-viable (**F**) cells on day 5 determined by trypan blue exclusion. Data are presented as mean ± SD from triplicate assays, and the experiments were repeated three times. Statistical significance was assessed by Student’s *t*-test (** *p* < 0.001).

To determine the extent to which the sealers activate MAPK signaling in MC3T3-E1 cells, phosphorylation of c-Jun, p38, and ERK was assessed by Western blot. Both ZOE and BC-induced phosphorylation of all three MAPKs, with higher levels observed in the ZOE group (Figure 3A). Because ZOE induced stronger MAPK phosphorylation while BC showed greater ARS staining, cellular responses were examined to determine whether initial cell behavior contributed to these differences. Phase-contrast microscopy showed poor spreading, rounding, and detachment of cells surrounding the ZOE pellet, whereas BC-treated cells maintained a fibroblast-like morphology (Figure 3B). Cell numbers around ZOE were significantly lower than those around BC at both day 4 and day 5, with little change across time points. In contrast, BC-treated cultures demonstrated an increase in cell number (Figure 3C,D). Trypan blue exclusion confirmed higher viability in the BC group and reduced viability in the ZOE group (Figure 3E,F). These early differences in viability were not evident in later cultures used for ARS staining, suggesting that reduced proliferation and viability near ZOE occurred primarily during the initial culture period.

**Figure 4:**
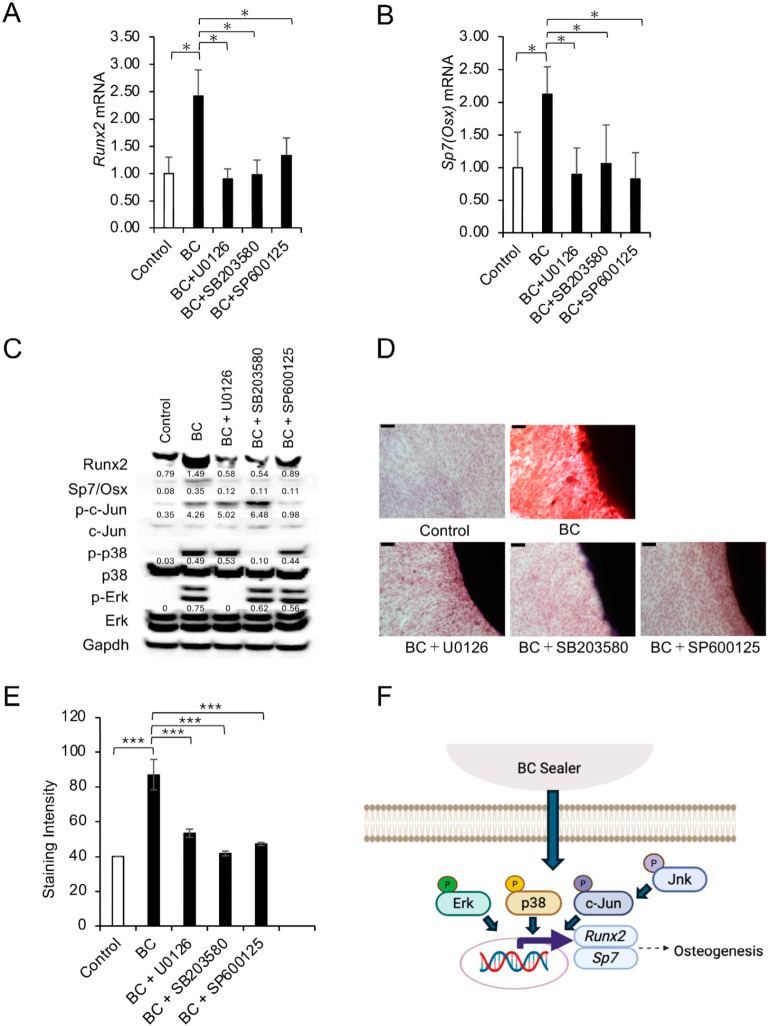
Effects of MAPK inhibition on BC-induced osteogenic differentiation in MC3T3-E1 cells. (**A**,**B**) qRT-PCR analysis of *Runx2* (**A**) and *Sp7* (*Osx*) (**B**) expression after 1 week of culture in OI medium under control conditions (no sealer), BC, or BC combined with MAPK inhibitors [U0126 (Erk), SB203580 (p38), SP600125 (Jnk)]. Expression levels were normalized to *Gapdh*. Data are presented as mean ± SD from triplicate assays, and the experiments were repeated three times. Statistical significance was assessed by one-way ANOVA with Tukey’s post hoc test (* *p* < 0.01). (**C**) Western blot analysis of Runx2, Sp7 (Osx), p-c-Jun, c-Jun, p-p38, p38, p-Erk, and Erk under the same conditions as (**A**). Gapdh (loading control). (**D**) Representative of ARS staining images of cells cultured with control (no sealer), BC, or BC plus MAPK inhibitors for 2 weeks with OIM. Scale bars = 100 μm. (**E**) Quantification of ARS staining intensity using ImageJ. Data are presented as mean ± SD from 5 fields, and the experiments were repeated three times. Statistical significance was assessed by one-way ANOVA with Tukey’s post hoc test (*** *p* < 0.0001). (**F**) Proposed schematic model, based on the present findings, illustrating that the BC sealer regulates MAPK signaling pathways (Jnk, p38, Erk) to induce osteogenic differentiation by modulating the expression of *Runx2* and *Sp7* (*Osx*) in pre-osteoblasts.

To investigate the role of MAPK signaling in BC-treated cells, ERK, p38, or JNK inhibitors were added to BC cultures. Inhibition of any of these pathways reduced *Runx2* and *Osx* mRNA levels (Figure 4A,B) as well as protein expression (Figure 4C), and significantly decreased ARS staining (Figure 4D,E). These findings indicate that MAPK signaling is involved in BC-associated osteogenic-related responses using this in vitro model (Figure 4F).

## 4. Discussion

Although overt chronic inflammation or tissue necrosis following sealer extrusion is infrequently reported, the absence of obvious clinical complications does not equate to biological neutrality [4,9,22,23]. Clinical outcomes do not reveal the underlying cellular stress responses, signaling dynamics, or differentiation processes occurring at the sealer–tissue interface [4,24,25]. Thus, defining how different sealers modulate viability and MAPK-mediated osteogenic-associated pathways provides essential mechanistic insight that complements clinical observations and helps clarify why materials with similar clinical profiles may behave differently at the molecular level.

This study examined the molecular and cellular responses of MC3T3-E1 pre-osteoblasts to four commonly used endodontic sealers and identified distinct patterns of MAPK activation, viability, and calcium-associated ARS staining. BC and Sealapex™ produced strong ARS staining, whereas ZOE and AH26^®^ showed minimal or no staining. Sealapex™ exhibited high solubility, leading to extensive calcium diffusion and inconsistent cell recovery, while AH26^®^ produced insufficient viable cells for molecular assays. Therefore, BC and ZOE, representing two widely used and chemically distinct sealers, were selected for mechanistic evaluation of MAPK signaling and osteogenic-associated molecular responses.

Calcium silicate-based BC sealers set through hydration and release calcium hydroxide, creating a highly alkaline environment and promoting hydroxyapatite formation [24]. These physicochemical properties, together with previously reported suppression of inflammatory cytokines and favorable clinical tissue responses, support the bioactive profile associated with BC [26,27,28]. ZOE sealers, while clinically established and valued for their handling properties, have been reported to demonstrate limited mineralization potential in vitro [4,9,25,29]. Building on these known characteristics, this study aimed to delineate the cellular and signaling mechanisms underlying the distinct osteogenic-associated profiles elicited by these materials.

Both BC and ZOE activated ERK, p38, and JNK pathways, highlighting that sealer exposure triggers broad MAPK signaling. ERK promotes early osteogenic differentiation through Runx2 phosphorylation [30], p38 supports later differentiation via Osx and ALP regulation [31,32], and JNK enhances Runx2 through c-Jun-mediated transcription [12,33,34]. In BC-treated cultures, inhibition of any of these pathways reduced *Runx2* and *Osx* expression and decreased ARS staining, suggesting that coordinated MAPK activation may contribute to BC-associated osteogenic responses. Previous reports suggest that ionic dissolution released from the BC sealer may stimulate the MAPK cascade, thereby promoting the expression of *Runx2* and *Osx* [16,33,35,36].

In this study, MAPK activation was assessed at 1 week, whereas ARS staining was performed at 2 weeks. The selected time points reflect the distinct temporal characteristics of intracellular signaling events and their downstream phenotypic outcomes. MAPK activation was assessed at 1 week because signaling responses, such as the phosphorylation of ERK, JNK, and p38, occur at relatively early stages following material exposure and precede transcriptional and phenotypic changes. In contrast, ARS staining was performed at 2 weeks, as calcium-associated mineral deposition represents a late-stage outcome of osteogenic differentiation. This process requires prolonged culture period under osteogenic conditions to become detectable and quantifiable. This time-course design is consistent with the biological sequence in which early signaling activation regulates subsequent gene expression and mineralization.

Interestingly, although ZOE induced stronger MAPK phosphorylation than BC, it did not enhance osteogenic-associated gene expression or ARS staining. This apparent paradox may be explained by the context-dependent and pathway-specific nature of MAPK signaling. MAPK activation is not uniformly pro-osteogenic; rather, the biological outcome depends on the specific MAPK branches involved, as well as the magnitude and duration of their activation [37,38]. Excessive or stress-dominant activation of p38 and JNK has been associated with impaired adhesion, reduced proliferation, and early apoptotic signaling rather than differentiation [38]. Consistent with this, ZOE transiently disrupted spreading, attachment, and viability during early culture, suggesting that MAPK hyperphosphorylation reflected an acute stress response. In contrast, BC appeared to induce a more balanced activation pattern permissive for survival and osteogenic-associated transcription. Thus, stronger MAPK activation alone is insufficient to drive osteogenesis; signaling quality and cellular context are critical determinants of differentiation outcomes.

Previous in vitro studies have demonstrated that calcium silicate-based materials modulate cellular behavior in a cell type-, concentration-, and exposure-dependent manner. A comprehensive review by Song et al. summarized that responses vary markedly among stem cells, osteoblasts, fibroblasts, and immune cells, and depend on exposure model, setting state, and ionic release profiles [28]. Within this context, the present findings using MC3T3-E1 cells represent a focused mechanistic evaluation of osteogenic-associated cellular responses under standardized in vitro conditions, rather than a comprehensive representation of all periapical cell types.

The solid-sealer model used in this study offers an important strength, as it closely mimics the clinical scenario in which a set sealer remains in direct contact with periapical tissues and exerts biological effects through surface-dependent interactions and localized ion release [4,24,25,39]. This approach allows evaluation of cellular responses and captures surface-dependent phenomena of the material in its clinically relevant form, capturing features that cannot be reproduced using extract-based or conditioned-media methods [28,33]. While inherent differences in curing behavior, solubility, and ion-release kinetics may introduce variability across materials, these characteristics reflect clinically relevant material properties.

Although the present study quantified calcium ion release and pH changes in the bulk immersion medium, spatial gradients at the immediate material–cell interface were not directly assessed. Future studies employing microscale analytical techniques would further clarify how localized physicochemical cues influence cell behavior. Comparative evaluation of solid versus extract models, inclusion of broader osteogenic markers, and assessment of apoptosis-related pathways in ZOE-treated cells would provide additional mechanistic insight. Ultimately, in vivo studies are necessary to determine how these signaling responses integrate within the complex periapical environment.

Importantly, recent in vivo evidence underscores the complexity of translating in vitro findings to tissue-level outcomes. Baldawa et al. reported that modified MTA formulations showed favorable histopathological responses, including reduced inflammation and enhanced dentinal bridge formation, in a rat pulpotomy model despite early inflammatory changes [40]. These findings indicate that transient cellular stress or cytotoxicity observed in vitro does not necessarily preclude favorable long-term tissue healing in vivo. Taken together, the present in vitro results provide mechanistic insight into sealer-induced cellular signaling and mineralization potential, which should be integrated with animal and clinical studies to more fully understand periapical tissue responses.

### Limitations

This study has several limitations that warrant consideration. Experiments were conducted using MC3T3-E1 cells, and responses may not fully reflect the complexity of human periapical tissues. ARS staining was used as an indicator of calcium-associated deposition and does not distinguish true matrix mineralization from calcium binding. Additional signaling pathways, including PI3K/Akt, mTOR, and Wnt/β-catenin, were not examined and may contribute to BC-associated responses [41,42,43,44]. Mechanistic analyses were limited to BC and ZOE, as Sealapex™ demonstrated high solubility and unstable material conditions consistent with prior ISO 6876-based reports [21,45], and AH26^®^ produced extensive cytotoxicity (approximately 90–100% non-viable fraction by day 4) with a large cell-free zone (~9 mm), precluding reproducible molecular analysis. Thus, findings should be interpreted within the context of the selected materials.

## 5. Conclusions

These findings highlight the importance of signaling context and cell viability in shaping osteogenic responses to endodontic sealers and provide mechanistic insight into the biological behavior of these materials. This information may aid in the interpretation of periapical tissue responses and in making more informed decisions when selecting sealers, particularly in cases where extrusion may occur.

## Data Availability

The original contributions presented in this study are included in the article and Supplementary Materials. Further inquiries can be directed to the corresponding author.

## Supplementary Materials

The following supporting information can be downloaded at: https://www.mdpi.com/article/10.3390/dj14030160/s1, Supplementary Figure S1: Effects of endodontic sealers on MC3T3-E1 cell viability. (supporting the exclusion of AH26^®^ from downstream molecular assays); Supplementary Figure S2: Calcium ion release and alkalinity generation by endodontic sealers.

## Abbreviations

The following abbreviations are used in this manuscript:

MAPK	Mitogen-activated protein kinase
ZOE	Zinc oxide eugenol
BC	Bioceramic
qRT-PCR	Quantitative reverse transcription PCR
ARS	Alizarin Red S
SD	Standard deviation
