# Supplementary material for: In Vitro Assessment of Osteogenic Modulation and Molecular Responses Induced by Contemporary Endodontic Sealers in MC3T3-E1 Pre-Osteoblasts
Source: Dent J (Basel). 2026 Mar 11;14(3):160. doi: 10.3390/dj14030160 (PMC13025870; doi:10.3390/dj14030160)
Supplement: Supplementary file 1 [file dentistry-14-00160-s001.zip › Figure S1.pdf]

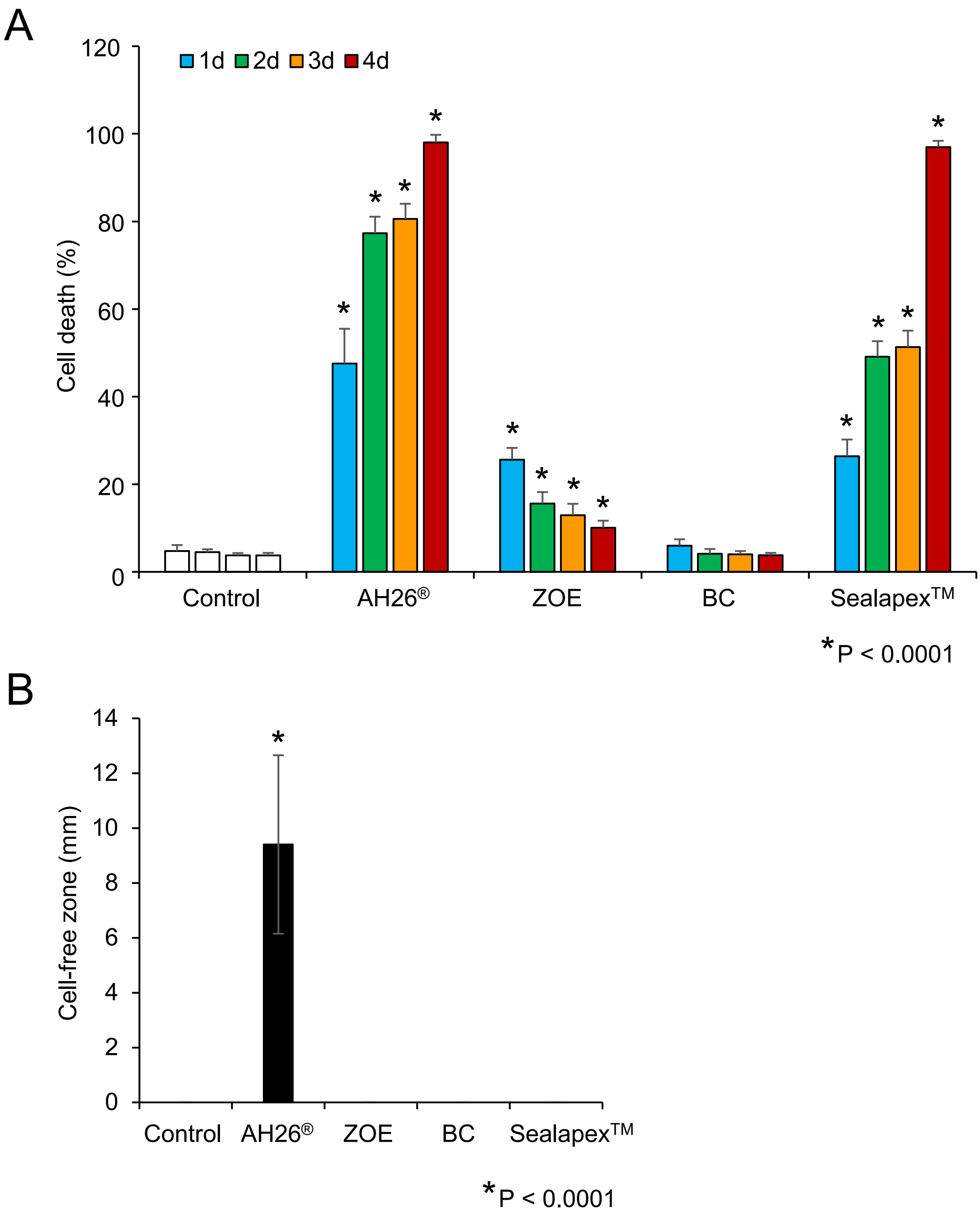

Figure S1. Effects of endodontic sealers on MC3T3-E1 cell viability. (a) Percentages of non-viable cells on day 1, day 2, day 3, and day 4 were determined by trypan blue exclusion. (b) Extent of the non-viable / cell-free zone (AH26®, Figure 1b) was quantified as the distance from the sealer pellet. Data are presented as mean ± SD from triplicate assays, and the experiments were repeated three times. Statistical significance was assessed by Student's t-test (*$p < 0.0001$).
