# Supplementary material for: In Vitro Assessment of Osteogenic Modulation and Molecular Responses Induced by Contemporary Endodontic Sealers in MC3T3-E1 Pre-Osteoblasts
Source: Dent J (Basel). 2026 Mar 11;14(3):160. doi: 10.3390/dj14030160 (PMC13025870; doi:10.3390/dj14030160)
Supplement: Supplementary file 1 [file dentistry-14-00160-s001.zip › Figure S2.pdf]

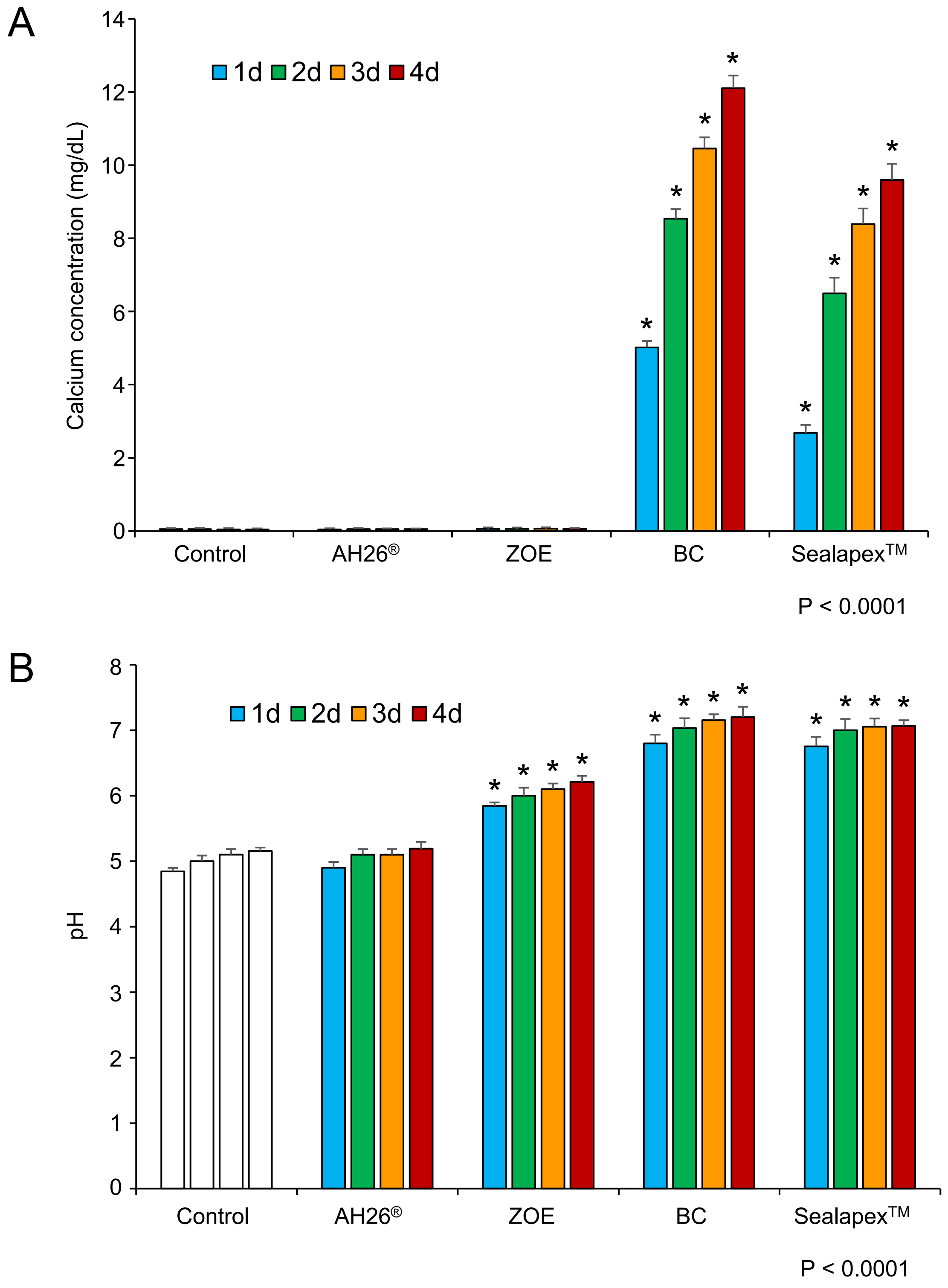

Figure S2. Calcium ion release and alkalinity generation by endodontic sealers. (a) Calcium release from endodontic sealers under cell-free conditions measured from day 1 to day 4. (b) pH changes induced by the sealers under the same conditions. Data are presented as mean ± SD from triplicate assays, and the experiments were repeated three times. Statistical significance was assessed by Student's t-test (*$p < 0.0001$).
